# Effect of Inconel 718 Filler on the Microstructure and Mechanical Properties of Inconel 690 Joint by Ultrasonic Frequency Pulse Assisted TIG Welding

**DOI:** 10.3390/ma17122857

**Published:** 2024-06-11

**Authors:** Ke Han, Xin Hu, Xinyue Zhang, Hao Chen, Jinping Liu, Xiaodong Zhang, Peng Chen, Hongliang Li, Yucheng Lei, Jinhui Xi

**Affiliations:** 1 School of Materials Science & Engineering, Jiangsu University, Zhenjiang 212013, China; 2 China Nuclear Power Engineering Co., Ltd., Beijing 100822, China; 3 Key Laboratory for Highly Efficient and Intelligent Welding of China National Nuclear Corporation, China Nuclear Industry 23 Construction Co., Ltd., Beijing 101300, China

**Keywords:** TIG welding, Inconel 690 alloy, Inconel 718 filler, microstructure, mechanical properties, ductility dip cracking

## Abstract

Ultrasonic frequency pulse assisted TIG welding (UFP-TIG) experiments were conducted to join Inconel 690 alloy (IN690) by adding Inconel 718 alloy (IN718) as the filler. The effect of the filler on the microstructure, mechanical properties, and ductility dip cracking (DDC) susceptibility of IN690 joints were investigated. The results show that a variety of precipitates, including MC-type carbide and Laves phases, are formed in the weld zone (WZ), which are uniformly dispersed in the interdendritic region and grain boundaries (GBs). The increase in the thickness of the IN718 filler facilitates the precipitation and growth of Laves phases and MC carbides. However, the formation of Laves phases in the WZ exhibits a lower bonding force with the matrix and deteriorates the tensile strength of IN690 joints. Due to the moderate content of Laves phases in the WZ, the IN690 joint with 1.0 mm filler reaches the maximum tensile strength (627 MPa), which is about 96.5% of that of the base metal (BM). The joint with 1.0 mm filler also achieves the highest elongation (35.4%). In addition, the strain-to-fracture tests indicate that the total length of cracks in the joint with the IN718 filler decreases by 66.49% under a 3.8% strain. As a result, the addition of the IN718 filler significantly improves the mechanical properties and DDC resistance of IN690 joints.

## 1. Introduction

As a new high chromium nickel-based alloy, Inconel 690 alloy (IN690) presents excellent high-temperature properties and corrosion resistance in the harsh nuclear power service environment [1]. IN690 alloy becomes the preferred material for heat transfer tubes in third-generation nuclear power steam generators [2]. Therefore, the high-quality welding process of IN690 alloy plays a pivotal role in the safety of nuclear power products. Due to the merit of minimal splash, simple operation, and low cost, tungsten inert gas (TIG) arc welding presents excellent application prospects for the welding of U-shaped heat transfer tubes in nuclear reaction steam generators with small tube diameters and thin tube walls. According to previous reports [3,4], the IN690 joint by TIG welding presents weak mechanical properties in the WZ and high crack (DDC) sensitivity. Therefore, high-quality TIG joining of IN690 alloy is essential to guarantee the safety of nuclear reaction steam generators.

So far, numerous studies have indicated that the addition of filler metal could restrain the susceptibility to DDC in the IN690 joint [4,5]. However, there is currently no consensus in the published literature regarding the formation mechanism of DDC in the IN690 joint. Several studies have highlighted that nickel-based alloys with tortuous GBs exhibit lower susceptibility to DDC compared with materials with flat GBs [6,7]. A few researchers have revealed that the addition of elements such as Nb and Mo elements induces a transition in the grain boundary morphology of IN690 alloy, further impacting its DDC sensitivity [4,5,8]. Rapetti et al. [4] summarized a correlation equation between the DDC sensitivity factor and alloy elements like Nb, C, and S. Ahn et al. [8] evaluated the DDC susceptibility of IN690 alloy by varying the Nb content in the electrodes. The reduction in Nb content led to the significant coarsening of M23C6 carbides at the GBs, contributing to crack propagation. Kadoi et al. [5] assessed the susceptibility to DDC cracking by strain-to-fracture tests (STFs) during laser welding of IN690 alloy. It has been demonstrated that the distribution of alloying elements in IN690 toward GBs and changes in grain boundary orientation also impact DDC sensitivity. Apart from the intergranular carbide precipitation in the nickel-based alloy, the grain size of WZ also has a significant effect on DDC susceptibility [9]. Hua et al. pointed out that the external ultrasonic field can refine the grain of FM–52 M welds and further decrease the detrimental GB length, resulting in improvement in DDC resistance [10].

Based on the above conclusions, Nb-rich IN 718 alloy as the filler metal and the UFP process are applied in the present work. The effect of IN718 thickness on the microstructure evolution, tensile properties, and DDC susceptibility of the IN690 joint is investigated in detail.

## 2. Experimental Methods

### 2.1. Welding Material

**Figure 1:**
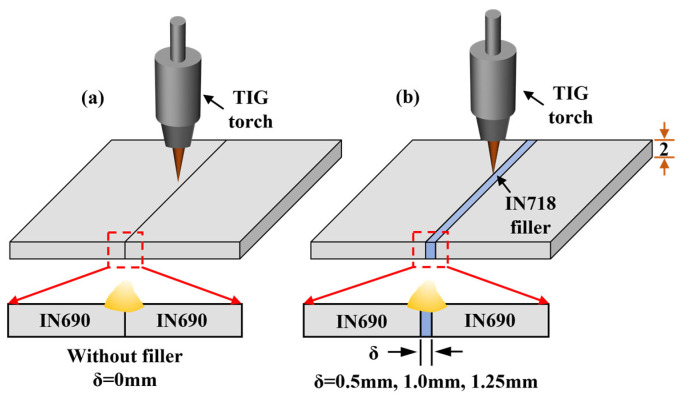
Schematic diagrams of TIG welding: (**a**) without filler; (**b**) with IN718 filler.

**Figure 2:**
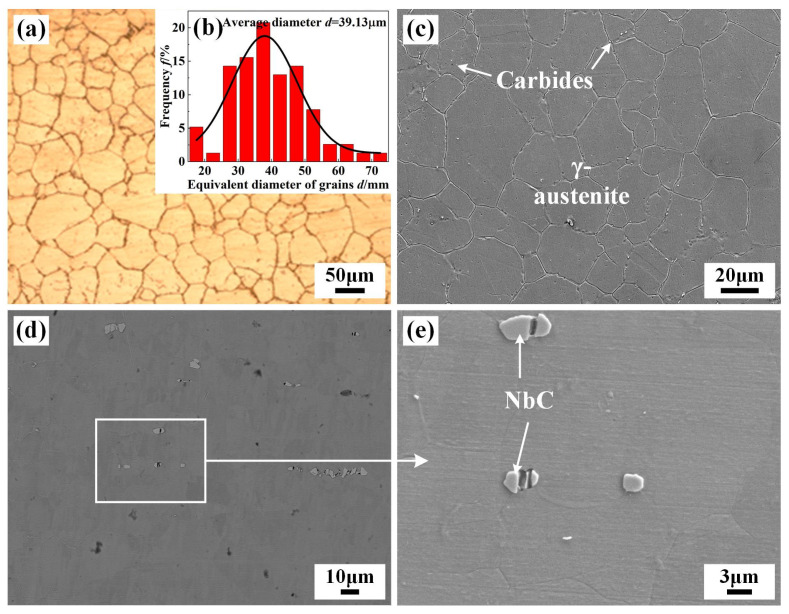
Microstructure and grain distribution of IN690 and IN718 alloys: (**a**) metallographic microstructure; (**b**) average grain size; (**c**) high-resolution image of IN690 alloy; (**d**) low-resolution and (**e**) high-resolution image of IN718 alloy.

Hot-rolled IN690 and IN718 alloys were used as the BM and filler metal. All the BMs and filler metals were prepared with a wire cutting machine. The thicknesses of the IN718 filler metal were 0.5 mm, 1.0 mm, and 1.25 mm, respectively. The schematic diagrams of TIG welding with and without filler are shown in Figure 1. The chemical composition of the IN690 and IN718 alloys are shown in Table 1. The microstructures of the BM and IN718 filler are shown in Figure 2. IN690 alloy was mainly equiaxed crystals with an average equivalent diameter of 39 μm, comprising γ-Ni and minor carbides distributed along the GBs. The IN718 filler presented a similar microstructure as the IN690 alloy. Due to the higher content of Nb element, blocky NbC carbides were precipitated.

A DC argon arc welding machine (model WSM-160, Aotai Electric Co., Ltd., Jinan, China) with direct current reversed polarity and a self-made ultrasonic excitation system were used to join the IN690 alloy. The TIG welding parameters and ultrasonic excitation parameters are shown in Table 2.

### 2.2. Microstructure Characterization

The joint was cut vertically along the weld seam direction, and the cross-section was used as an observation surface after being polished with sandpaper (layer by layer, 80#→240#→600#→800#→1200#→2000#) and diamond paste with a particle size of 0.5 µm. Electrolytic corrosion was performed using a 10% chromate solution (CrO_3_). An optical microscope (OM, Observer. Z1M Zeiss, Oberkohen, Germany) was used to observe the microstructure of different regions in the sample. The high-resolution microstructure and fracture morphology of the IN690 joint were characterized by an NovaNano 450 field emission scanning electron microscope (SEM, FEI Company, Hillsboro, OR, USA). SEM was also used to detect the element types, contents, and distributions of precipitates in the joint. Electron backscatter diffraction (EBSD) was applied to characterize the GB features of WZ in the IN690 joint.

### 2.3. Mechanical Properties Test

The microhardness of the IN690 joint was measured from the BM to the WZ by an FM-ARS900 automatic microhardness tester (Future-tech Corp., Kanagawa, Japan) with a load of 300 gf, loading time of 15 s, and distance between indents of 0.5 mm. An RDL100 electronic universal testing machine (Sinotest Equipment Co., LTD., Changchun, China) was applied to characterize the tensile properties (tensile strength, yield strength, and elongation), with a tensile rate of 2 mm/min. Strain-to-fracture tests (STFs) were carried out by a Gleeble 3500 thermal simulation tester (Dynamic Systems Inc., New York, NY, USA) to investigate the DDC sensitivity of the IN690 joint. The test was carried out in an argon atmosphere with a heating rate of 100 °C/s and a holding time of 10 s before stretching. The displacement rate was set to 0.1 mm/s for the required strain amount. The morphology and number of cracks formed in the IN690 joint were observed and counted by OM.

## 3. Results and Discussion

### 3.1. Effect of IN718 Filler on Microstructure

**Figure 3:**
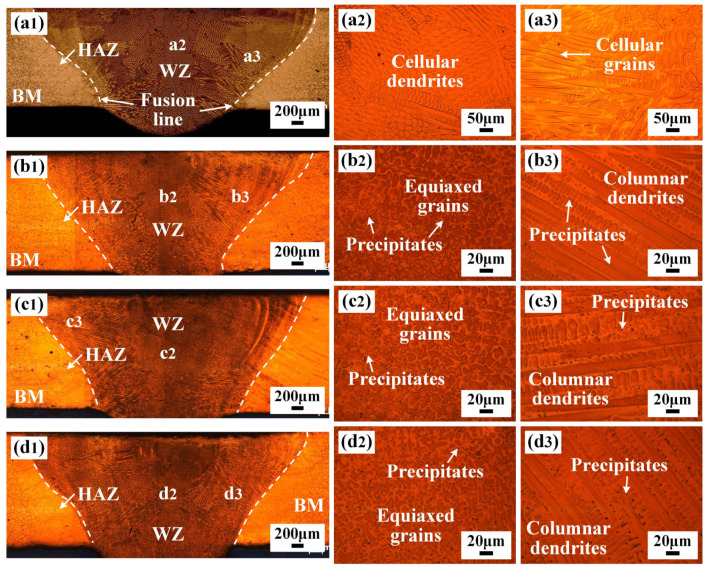
Weld morphology and microstructure of IN690 joints with different thickness IN718 filler: (**a1**–**a3**) without filler; (**b1**–**b3**) 0.5 mm; (**c1**–**c3**) 1.0 mm; (**d1**–**d3**) 1.25 mm.

The cross-section morphologies and microstructure of the IN690 joint with the IN718 filler are shown in Figure 3. No pores were found in the joint. The melting pool of the IN690 joint presented a typical bowl-like morphology. Due to the different constitutional supercooling in the WZ, the grain morphology of different regions in the WZ also varied. For the traditional IN690 joint (1#), the microstructures in the middle and edge regions of WZ were mainly composed of cellular dendrites and cellular grains, which corresponded to the reported results [11]. When the IN718 filler metal was introduced, the content of the alloy elements in the WZ increased. Therefore, the microstructures in the middle and edge regions of WZ presented typical equiaxed grains and columnar dendrites. With the increase in the thickness of IN718 filler, the primary dendrite arm spacing (PDAS) and secondary dendrite arm spacing (SDAS) decreased gradually. The PDAS decreased from 18.5 µm to 12.3 µm and the SDAS decreased from 8.5 µm to 4.6 µm. In addition, a few black precipitates were observed in the cross-section of the IN690 joint, which were mainly scattered in the interdendritic regions. As the thickness of the IN718 filler metal increased, the volume of the precipitates also increased, as shown in Figure 3(b3–d3). Moreover, notable grain coarsening was observed in the HAZ, which was due to the welding thermal cycle [12]. The abnormal grain growth gave rise to the local softening of HAZ.

**Figure 4:**
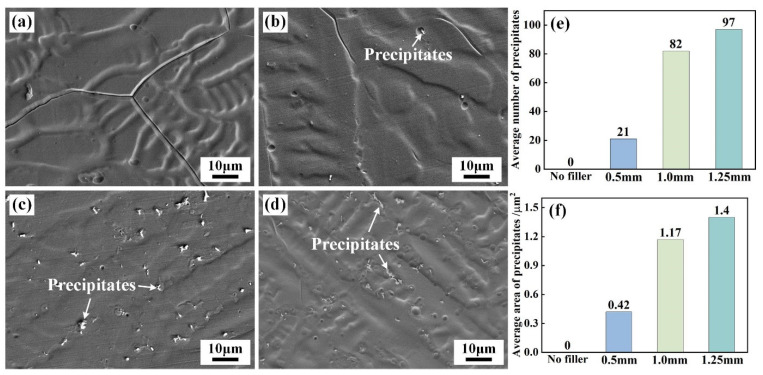
Morphology and content of precipitates in the WZ of IN690 joint with different thickness IN718 fillers: (**a**) without filler; (**b**) 0.5 mm; (**c**) 1.0 mm; (**d**) 1.25 mm. (**e**) Average number; (**f**) average area.

Figure 4 shows the morphologies of the precipitates in the WZ of the IN690 joint with different thickness IN718 layers. Compared with joint 1#, the addition of IN718 layers facilitated the precipitation of white precipitates. Only a few punctate precipitates were distributed in IN690 joint 2#. However, when the thickness of IN718 filler exceeded 1.0 mm, the IN690 joint comprised a large size and irregular precipitates. The average number and area of precipitates in the WZ were counted and the statistical results are shown in Figure 4e,f. The average number of precipitates increased from 21 to 97, and the average area of precipitates increased from 0.42 µm^2^ to 1.40 µm^2^ as the thickness of the IN718 filler changed from 0.5 mm to 1.0 mm.

**Figure 5:**
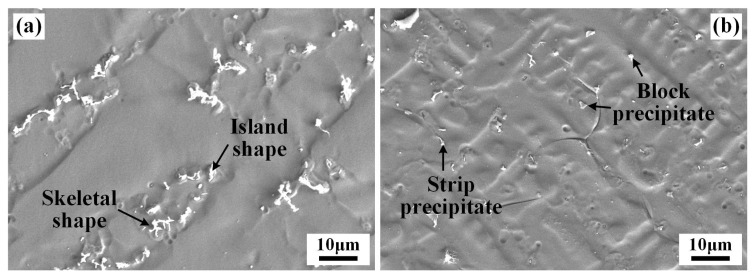
Morphologies of precipitates in the WZ of joint 3#. (**a**) skeleton and island shape; (**b**) strip, and block shape.

The addition of the IN718 filler had an important influence on the morphologies of precipitates in the WZ of the IN690 joint, as shown in Figure 5. For joint 2#, multiple-shape precipitates, including skeleton, island, strip, and block, were formed in the WZ. The content of skeleton-like and island-like precipitates was dominant, which were mainly distributed in the interdendritic regions. According to the previous report [13], the precipitates in the interdendritic regions presumably were Laves phases and carbides.

**Figure 6:**
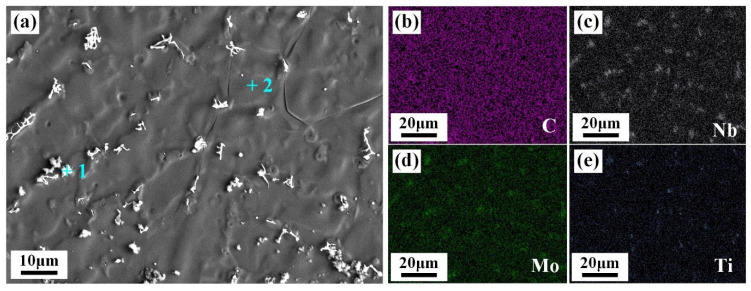
SEM image and EDS map (**a**) and scanning of the precipitates in the interdendritic regions of WZ (joint 3#); (**b**) C; (**c**) Nb; (**d**) Mo; (**e**) Ti.

The elemental distribution of mapping scanning and line scanning analysis was applied to verify the chemical composition of the precipitates, as shown in Figure 6. The irregular precipitates were enriched in Nb and Mo elements. Zhang et al. [14] and Wen et al. [15] reported that Nb-rich precipitates were Laves phases resulting from the eutectic reaction L → γ + Laves. It is well known that Laves phases are A_2_B-type phases. A expresses the alloy elements Ni, Cr, Fe, and Co, while B mainly denotes the alloy elements Nb and Ti. As seen from the results in Table 3, the content of Cr, Ni, and Fe elements in the irregular precipitates (+1) was significantly higher than that of Nb, Ti, and Mo elements, therefore further proving that the irregular precipitates were (Cr,Ni,Fe)_2_(Nb,Ti,Mo)-type Laves phases. The formation of Laves phases was due to Nb element segregation in the interdendritic region during the welding cooling process. The addition of the IN718 filler provided Nb atoms in the WZ. As a typical positive segregation element, the Nb element presents serious segregation behavior in the last stage of solidification [13,16]. Therefore, Nb-rich Laves phases are formed in the interdendritic regions. The morphology of the Laves phases also has a large influence on the tensile properties of the joint [17]. Irregular Laves phases present a lower bonding force with the matrix and are more prone to breakage and debonding failure when subjected to the applied loads [18]. Therefore, the formation of Laves phases with complex shapes would deteriorate the mechanical properties of the IN690 joint.

**Figure 7:**
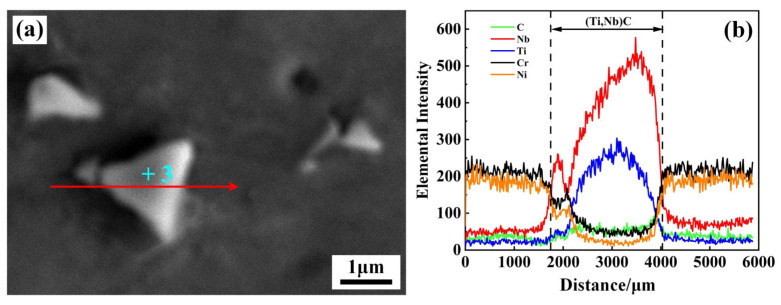
SEM morphology and elemental distribution of MC carbides in joint 3#: (**a**) SEM morphology; (**b**) line scanning data.

Apart from the formed Laves phases, block precipitates also appeared in the interdendritic and GB regions. Figure 7 shows the morphology and elemental distribution of the block precipitates. As seen in Figure 7, there was a sudden increase in the content of Nb and Ti elements from the matrix to the block precipitates. The EDS results (+3) in Table 3 indicate that the atom fractions of Nb and Ti elements were 21.61% and 28.55%, respectively. Han et al. [19] and Qin et al. [20] pointed out that Ti, Nb, and Ta elements are the forming elements of the MC-type carbide in nickel-based alloy. As a result, the block precipitates were presumed to be (Ti,Nb)C, abbreviated as an MC-type carbide. As the thickness of the filler increased, the content of Nb element in the weld gradually increased and the ratio of C/Nb in the liquid phase decreased, causing a portion of MC-type carbides, such as NbC, to transform into the Laves phase [15].

**Figure 8:**
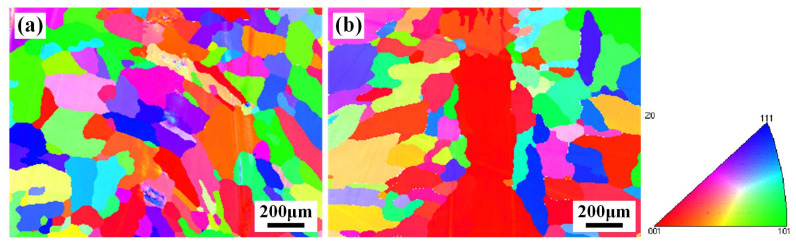
Crystallographic orientation maps of WZ in joints 1# (**a**) and 3# (**b**).

**Figure 9:**
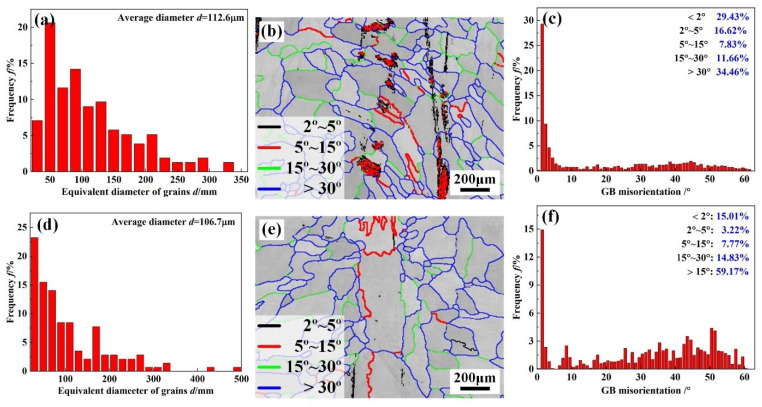
Grain size statistical histograms, GB misorientation maps, and GB misorientation statistical histograms of the WZ in joints 1# (**a**–**c**) and 3# (**d**–**f**).

Figure 8 shows the grain morphologies and orientation of WZ in joints 1# and 3#. As seen from Figure 8, the dominated orientations of grains in the WZ of joints 1# and 3# were parallel with <001> direction. As for nickel-based polycrystalline alloys, grains with <100> orientation provide a plane along the direction of dendritic growth that is easy for crack propagation [21]. Statistical data on the equivalent circular diameter of the grains and GB misorientation in the WZ are shown in Figure 9. The grain sizes in the WZ of joints 1# and 3# were 112.6 μm and 106.7 μm, respectively, which was ascribed to the precipitation of the Laves phases and MC carbides. Schneider et al. found that the Laves and NbC phases distributed along the GBs could refine the grain of WZ by pinning the GBs [22]. However, the content of precipitates in the WZ of joint 3# was lower, causing a limited grain-refinement effect. The WZ of joint 1# was mainly low-angle GBs (LAGBs, <15°), with a fraction of 53.88% (Figure 9c), while the addition of the IN718 filler facilitated the transformation GB angle of WZ from LAGBs to high-angle GBs (HAGBs). The fraction of HAGBs with a misorientation angle > 15° in joint 3# was approximately 74% (Figure 9f). The density of LAGB can be an indicator of the magnitude level of local deformation or internal residual stress [23]. The higher fractions of LAGBs indicated that the WZ of joint 1# presented larger internal residual stress, making it easy to induce the propagation of cracks. Therefore, it can be hypothesized that the IN690 joint with the addition of the IN718 filler exhibited excellent mechanical properties and crack resistance.

### 3.2. Effect of IN718 Filler on the Mechanical Properties of IN690 Joints

**Figure 10:**
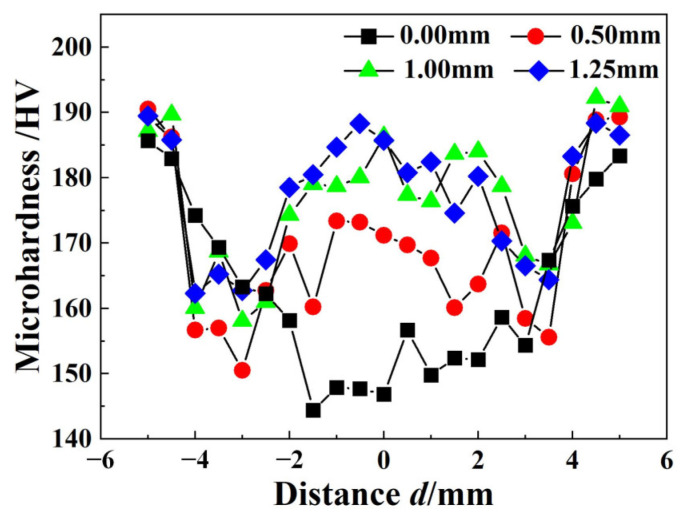
Microhardness distribution of IN690 joints with different thickness filler.

The microhardness distribution of the IN690 joint with different thickness fillers are shown in Figure 10. For joint 1#, the microhardness of the joint evidently decreased from the BM to the WZ. As shown in Figure 3 and Figure 8, the grain size in the WZ was higher than that in the BM and HAZ, resulting in the notable softening of the WZ. On the other hand, no precipitates were produced in the WZ. Therefore, the WZ in joint 1# presented minimum microhardness. However, the addition of the IN718 filler changed the microhardness distribution of the IN690 joint. The microhardness of IN690 joints with the IN718 filler demonstrated a W-shape distribution, and the lowest microhardness appeared in the HAZ. For the solid solution strengthened nickel-based alloys (IN690 alloy), the grain size showed an important influence on the mechanical properties of the joint. Compared with the BM, the thermal cycle resulted in the significant coarsening of grains in the HAZ and WZ. According to the Hall–Petch relationship [24], the BM presented the highest microhardness in the IN690 joints. With the increased thickness of the IN718 filler, the microhardness of the WZ gradually increased, decided by the formation of precipitates. The presence of the Laves phase and MC carbide could pin the GB and impede the dislocation movement during the deformation process [25]. As shown in Figure 4, the increase in the thickness of the IN718 filler brought about the increased content of precipitates and obtained a better strengthening effect. On the other hand, Ye et al. reported that ultrasonic frequency pulse promoted the oscillation of melt pool under the effect of cavitation and acoustic streaming, resulting in the refinement and uniform distribution of the precipitates [26]. Therefore, as the thickness of the filler increased, the microhardness of the WZ obviously increased.

**Figure 11:**
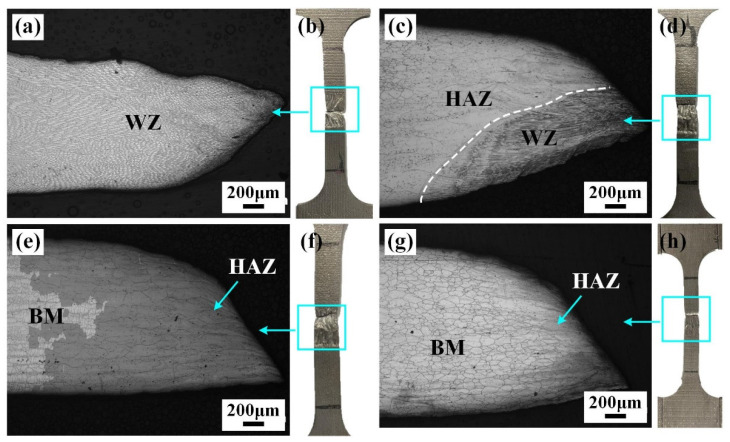
Fracture position of IN690 joints with different thickness filler: (**a**,**b**) without filler; (**c**,**d**) 0.5 mm; (**e**,**f**) 1.0 mm; (**g**,**h**) 1.25 mm.

**Figure 12:**
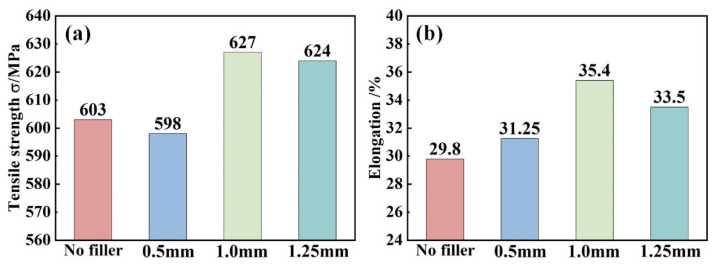
Effect of filler thickness on the mechanical properties of IN690 joints: (**a**) tensile strength; (**b**) elongation.

Figure 11 and Figure 12 show the fracture location and tensile properties of IN690 joints. It was obvious that all the joints fractured along the different regions. Due to the lowest microhardness in the WZ, joint 1# fractured at the WZ (Figure 11a). When the thickness of the IN718 filler was 0.5 mm, joint 1# fractured at the WZ. However, when the thickness of the IN718 filler was over 1.0 mm, the fracture position of the joints shifted to the HAZ. This was related to the precipitation strengthening effect resulting from the formation of the Laves phases and MC carbides. With the increase in IN718 filler thickness, the tensile strength and elongation of the IN690 joint firstly increased and then decreased. As shown in Figure 4, the increase in filler thickness caused the increased content of precipitates (Laves phases and carbides), resulting in an obvious strengthening effect. Therefore, the tensile strength of the IN690 joint with 1.0 mm thick filler reached 96.5% of that of the IN690 BM. When the thickness of filler further increased, the content of the Laves phases dominated in the WZ. The weak bonding force between the Laves phases and the matrix contributed to the debonding failure during the deformation process, which weakened the mechanical properties of the IN690 joint.

**Figure 13:**
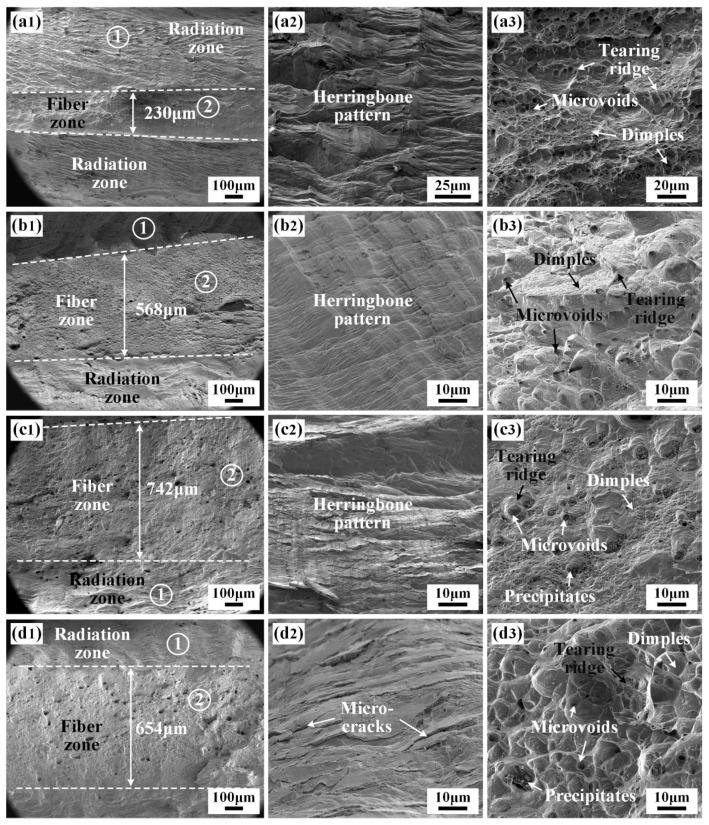
Fracture morphology of IN690 joints with different thickness filler: (**a1**–**a3**) without filler; (**b1**–**b3**) 0.5 mm; (**c1**–**c3**) 1.0 mm; (**d1**–**d3**) 1.25 mm.

The fracture morphologies of the joints are shown in Figure 13. All the fracture surfaces were composed of a fiber zone and a radiation zone. The fracture features in the fiber zone and radiation zone were mainly the deformed dimples and radial herringbone patterns, respectively, as shown in Figure 13. As the thickness of IN718 filler increased, the width of the fiber zone in the fracture surface firstly increased and then decreased, indicating that joint 3# displayed the best ductility and toughness. A large number of dimples were visible in the fiber zone of the fracture surface (Figure 13(a3,b3,c3,d3)). As the thickness of the filler increased, the formed dimples became larger and deeper, and the numbers of distinct precipitates at the bottom of the dimples in joints 3# and 4# were higher than that of joint 1#. In addition, the nucleation and propagation paths of cracks in the WZ during the tensile deformation process were affected by the morphology, size, and distribution of the precipitates. Zhao et al. found that precipitates with a moderate size and number could relax the stress concentration at the crack tip, effectively hinder crack extension, and improve the fracture toughness of the BM [27]. The large and deep dimples were present in the joints with a thinner filler. Once the thickness of the IN718 filler was 1.25 mm, the formation of the brittle Laves phases exacerbated the ductility of the IN690 joint, bringing about the decrease in the width of the fiber zone. Therefore, joint 3# presented sound mechanical properties.

### 3.3. Effect of IN718 Filler on the DDC Susceptibility of IN690 Joints

**Figure 14:**
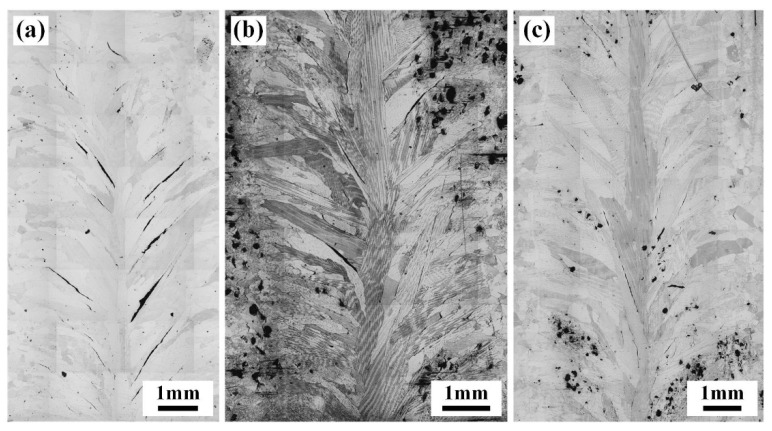
Macroscopic morphology of the IN690 joints under different strain variables: (**a**) 3.8%; (**b**) 3.8%; (**c**) 6%.

**Figure 15:**
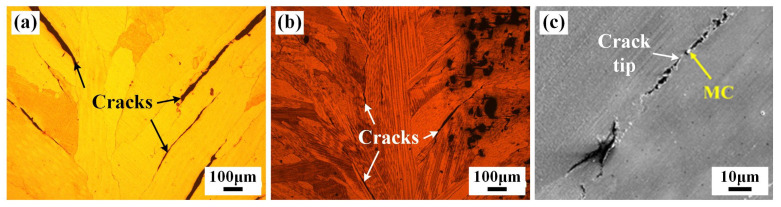
Formed crack features in joints 1# (**a**) and 3# (**b**,**c**) with 3.8% tensile strain.

The strain-to-fracture (STF) test is recognized as an effective analytical method to evaluate susceptibility to DDC [28]. The morphology, number, and length of cracks in the joints subjected to the STF test (1000 °C) are shown in Figure 14 and Table 4. After STF tests, a large number of cracks appeared in the WZ of the IN690 joint. All the cracks propagated along the GBs, especially for the coarse columnar grains. The statistics results in Table 4 manifest that the number and total length of cracks in joint 3# with the 3.8% strain were considerably lower than that of joint 1#. Increasing the strain level to 6%, the total crack length in joint 3# was 21,723.5 µm, which was significantly decreased compared with joint 1#. As seen in Figure 15, the cracks propagated along the long and straight GBs in joint 1#. Wei et al. found that the long and straight GBs slid easily to aggravate crack propagation during deformation, especially along the direction perpendicular to the loading direction [29]. Therefore, joint 1# presented a higher DDC susceptibility. When introducing the IN718 filler, Nb-rich precipitates were produced along the GBs. On the one hand, Nb-rich precipitates could pin the migrated GBs, causing an increase in GB tortuosity [4]. On the other hand, Ramirez et al. reported that the formation of Nb-rich precipitates resulted in a general increase in the threshold strain required to initiate cracking during the STF test [30]. As a result, only sparser and smaller cracks were observed in IN690 joint 3# under tensile strain. It was demonstrated that the introduction of the IN718 filler could effectively improve susceptibility to DDC.

## 4. Conclusions

In this work, the IN718 filler was introduced to join the IN690 alloy. The effect of the IN718 filler thickness on the microstructure, mechanical properties, and DDC susceptibility of the IN690 joint by UFP-TIG were systematically studied. Conclusions were drawn as follows:

(1) After introducing the IN718 filler, a large number of precipitates, including MC-type carbides and Laves phases, are formed in the interdendritic regions of the WZ, which is related to the serious segregation of Nb elements. With the increase in filler thickness, the content of Laves phases and carbides increases significantly.

(2) Due to the HAZ grain coarsening and the formation of precipitates in the WZ, the microhardness of the WZ is higher than that of the HAZ. With the increased thickness in IN718 filler, the microhardness and tensile properties of the IN690 joint are promoted. Joint 3# presents an exceptional tensile strength (627 MPa) and elongation (35.4%), about 4% and 18.8% higher than that of joint 1#.

(3) Compared with joint 1#, the total length of cracks in joint 3# decreases by 66.49% under the effect of a 3.8% strain. Elevated strains up to 6% still result in a lower crack number and length than that of joint 1#, closely related to the presence of precipitates at the GBs. Therefore, the addition of the IN718 filler significantly improves the DDC resistance of the IN690 joint.

## Figures and Tables

**Table 1:** Chemical composition (wt.%) of IN690 and IN718 alloys.

Alloy	Alloy Element											
	Ni	Cr	Fe	Mo	Nb	Al	Ti	C	Mn	Si	Cu	S
IN 690	60.02	30.39	8.88	-	-	0.2	0.21	0.02	0.197	0.07	0.01	0.002
IN 718	53.90	18.01	17.99	3.07	5.32	0.42	1.02	0.08	0.07	0.09	0.02	0.001

**Table 2:** Welding parameters and ultrasonic excitation parameters.

Welding Number	Thickness of Filler (mm)	Welding Current (A)	Voltage (V)	Welding Velocity (mm·min^−1^)	Shield Gas Flow (L·min^−1^)		Excitation Voltage (V)	Excitation Frequency (kHz)
					Top Surface	Back Surface		
1#	0 (without filler)	52	12~12.5	100	15	18	60	60
2#	0.5							
3#	1.0							
4#	1.25							

**Table 3:** EDS results of white precipitates in WZ (joint 1#, at.%).

Regions	Alloy Elements (%)							Possible Phases
	Ni	Cr	Fe	Nb	Ti	C	Mo	
+1	34.13	20.76	7.52	25.86	5.99	0	5.74	Laves phases
+2	9.18	7.22	3.53	32.25	9.67	36.84	1.31	γ-Ni matrix
+3	4.68	5.88	2.70	21.61	28.55	35.08	0.68	MC-type carbide

**Table 4:** Statistics results of cracks in Figure 15.

Group	Strain/%	Number	Total Length/µm	Average Length/µm	Maximum Length/µm
Joint 1#	3.8%	29	33,973.49	1171.50	3666.80
Joint 3#	3.8%	19	11,385.00	599.21	1612.13
	6%	20	21,723.50	1068.18	2599.70

## Data Availability

The raw/processed data required to reproduce these findings cannot be shared at this time due to technical or time limitations.
